# Multicenter survey about leg length discrepancy and total hip arthroplasty: preoperative and intraoperative management

**DOI:** 10.1007/s12306-024-00837-x

**Published:** 2024-07-05

**Authors:** Davide Stimolo, Salvatore Lo Giudice, Fabrizio Matassi, Matteo Innocenti, Roberto Civinini, Filippo Boniforti

**Affiliations:** 1 University of Florence, School of Human Health Sciences, Largo Brambilla, 3 Florence 50134, Italy; 2 Department of Orthopaedics and Traumatology, AOU Careggi, Largo Piero Palagi 1, 50139 Florence, Italy; 3 AOUP Paolo Giaccone Palermo, University of Palermo, Via del Vespro 129, 90127 Palermo, Italy; 4 Fondazione Istituto G. Giglio, Cefalù, Contrada Pietra Pollastra, 90015 Cefalù, Italy

**Keywords:** Leg length discrepancy, LLD, Total hip arthroplasty, Complications, Survey

## Abstract

**Background:**

We created a multicenter survey for Italian orthopedic surgeons on how they approach leg length discrepancy (LLD) when dealing with primary total hip arthroplasty. Aim of the study was to show how surgeons manage LLD and follow the literature recommendations during clinical practice.

**Methods:**

The survey was composed of 25 questions divided into four sections: 1—surgeon’s profile, 2—preoperative and 3—intraoperative evaluation, and 4—postoperative management. In this paper, we report results to answer Sects. 1 and 2. Absolute and relative frequencies of answers to Sects. 2 and 3 are reported. We divided the participants in subgroups based on the “surgeon’s profile” and evaluated difference in the answers given.

**Results:**

Absolute and relative frequencies demonstrate low agreement among participants in all phases of LLD management. We demonstrated a statistically significant difference based on the surgeon’s profile regarding these questions: radiographic measure of LLD depending on working experience, *p* = 0.008; digital planning based on surgeons’ age, *p* < 0.001, and workplace, *p* = 0.026; intraoperative anatomical landmarks based on numbers of procedures per year, *p* = 0.020; and use of intraoperative X-rays based on working experience, *p* = 0.002.

**Conclusions:**

LLD is a debated topic with no definitive recommendations. Many decisions still depend on tradition and surgeons’ preference.

## Background

Total hip arthroplasty (THA) influences leg length and may determine leg length discrepancy (LLD) [1–3]. This may be a source of symptoms such as low back pain or limping, reducing overall outcomes. Treatment can be conservative or surgical [4]. It is crucial to evaluate patients before THA to identify LLD, use intraoperative landmarks to check leg length, and manage symptoms if LLD occurs after THA. Faldini [5] classifies patients by their preoperative risk factors for LLD. Low risk: Patients feel symmetrical, have no spinal or pelvic anatomical changes, and show less than 1 cm of limb length discrepancy. High risk: I) Patients with preoperative LLD because it is challenging to restore symmetry without raising the risk of dislocation; II) extra-articular causes of shortening as addressing the LLD at the joint level may not restore the natural articular geometry, and III) functional LLD due to muscular contractures, particularly with constrained hip abduction with adduction because patients with this condition do not tolerate leg lengthening well. Several methods for measuring clinical and radiographic lower limbs inequality have been described in the literature, but none is clearly more reliable than others [3]. Authors have also described intraoperative techniques to avoid LLD. There are also recommendations on the management of LLD, but no studies provide high level of evidence, and clinical practice is not always consistent. Furthermore, the introduction of new technologies in THA is challenging traditional techniques. Intraoperative navigation systems and robotic-assisted surgery aim to improve cup positioning and offer precise, real-time monitoring of leg length discrepancy (LLD) [6, 7]. Additionally, artificial intelligence (AI) is expected to be integrated into preoperative measurements of LLD in the near future [8]. This integration could lead to more accurate planning and better surgical outcomes.

We conducted a multicenter survey among Italian orthopedic surgeons to understand how they approach LLD when dealing with primary THA. The aim of this study is to show how surgeons manage LLD during preoperative evaluation and their preferred intraoperative techniques to ensure proper leg length.

## Materials and methods

We have created a survey titled “Leg length discrepancy after total hip arthroplasty: survey to orthopaedics surgeons” on Google Forms (Google, Mountain View, California, USA), with 25 closed questions, in four sections. In the first section, we identified the participants’ working profile (Table 1). In the second section, we asked about preoperative evaluation: clinical and radiographic LLD measurement, if they template preoperatively and if they make it digital or analog, if the surgical exposure can influence the accuracy of procedure (Table 2). The third section was dedicated to intraoperative techniques to avoid LLD: anatomical landmarks used, intraoperative X-rays, stability of components, and threshold of LLD accepted at the end of the operation (Table 3). In the fourth section, we asked about management of LLD after THA implant; however, this is not the object of this paper. We have invited colleagues from the Institutions of the Authors', including AOU Careggi–University of Florence, AOUP Paolo Giaccone–University of Palermo, and Fondazione Istituto G.Giglio—Cefalù, and members of the ASOTO (Associazione Siciliana di Ortopedia e Traumatologia Ospedaliera) to participate in our survey. Every participant answered on voluntary basis and in anonymous form. We shared the questionnaire by email or by WhatsApp (WhatsApp LCC), and after four weeks, we collected the answers. Only one option out of the given could be selected. We have analyzed only fully completed questionnaires and reported absolute and relative frequency of all the answers. Then we created subgroups based on different surgeon’s profile and matched it to LLD management. Subgroups have been: age < 35 or > 45; years of experience: < 10 or > 10; university hospital or not; area of expertise: orthopedic physicians (OP), trauma surgeons (TR), and lower limb replacement surgeons (RS); number of procedures per year: < 25 or > 25; and surgical exposure: anterior-based or postero-lateral. We analyzed the probability to give different answers by different subgroup membership. All independent and dependent variables are categorical and presented as absolute and relative frequencies. The association between them was tested with Fisher’s exact test and Chi-square test. Logistic regression was performed to assess the risk to answer correctly by area of expertise using the OP group as reference. All the analyses were performed using STATA software (version 17; StatCorp, College Station, TX, USA). An alpha level of 0.05 was considered significant. Ethics Committees of the main Institution (Careggi University Hospital, Florence) determined that no ethical approval was required, given that no patients were involved and answers to the questionnaire were completely anonymous, and since it was not possible to trace the personal data or email addresses of the survey participants.

**Table 1:** Section "Background" questions

Surgeon’s profile				
Age	< 35	35–45	45–60	> 60
Years of experience	Resident	0–10 years	10–20 years	> 20 years
Hospital of provenience	University hospital	I–II level	III level—Hub	Private hospital
Area of expertise	Trauma surgeon	Recon surgeon	Orthopedic physician	Others
Procedures per year	< 25	25–70	> 70	
Surgical approach	Anterior	Antero-lateral	Direct lateral	Postero-lateral

**Table 2:** Section "Materials and methods" questions

Preoperative evaluation				
How do you measure LLD clinically?	U-MM distance	ASIS-MM distance	Standing with graduated blocks under the shorter leg	Other
How do you measure LLD on X-rays?	LT-BIS distance	LT-IT distance	Standing long-leg X-rays	Other
Do you execute templating?	Never	Only for neck fractures	Only for elective surgery	Always
Digital or analog template?	Analog	Digital	I do not template	
Do you believe surgical approach can influence final LLD?	Yes	No		

*LLD* Leg length discrepancy, *U-MM* Umbilicus–medial malleolus, *ASIS-MM* Anterior–superior iliac spine–medial malleolus, *LT-BIS* Lesser trochanter–bisischiatic line, *LT-IT* Lesser trochanter–interteardrop line

**Table 3:** Section "Results" questions

Intraoperative evaluation					
Which one of these anatomical landmarks do you use to control intraoperative lengthening?	Comparison with contralateral leg	Comparison with preoperative template measurements	Distance between lesser trochanter and tip of the trial stem	Distance between great trochanter and tip of trial stem	Others
Do you execute intraoperative X-rays?	No	Yes	Only when in doubts		
After reduction with trial components, the prosthesis appears unstable. What do you do?	Cup evaluation (version, inclination)	Implant of longer head and accept eventual LLD	Implant of lateralizing neck of the stem, increasing femoral offset	Implant of the stem, few millimeters floating, increasing OF and LLD (especially cemented stems)	
Acceptable LLD at the end of operation	< 5 mm	5–10 mm	10–20 mm	> 20 mm	

*LLD* Leg length discrepancy, *OF* Offset

## Results

We have invited more than 200 orthopedic surgeons to participate in survey. After four weeks, we collected 109 answers. Of these, 104 have been analyzed because they were correctly completed. From Tables 4, 5, and 6, we have described absolute and relative frequency of the answers to each question. Five questions received more than 70% of agreement on one of the possible answers. Of these, only one in the Sects. "Materials and methods" and "Results:" The 83.7% of participants measure LLD clinically by the anterior–superior iliac spine–medial malleolus distance (ASIS-MM).

**Table 4:** Answers to Sect. "Background"

Surgeon’s profile				
Age	< 35	35–45	45–60	> 60
	**39 (37,5%)**	**18 (17,3%)**	**25 (24%)**	**22 (21,2%)**
Years of experience	Resident	0–10 y	10–20 y	> 20 y
	**34 (32,7%)**	**16,3%)**	**14 (13,5%)**	**39 (37,5%)**
Hospital of provenience	University hospital	I–II level	III level—Hub	Private hospital
	**42 (40,4%)**	**28 (26,9%)**	**10 (9,6%)**	**24 (23,1%)**
Area of expertise	TR	RS	OP	Others
	**36 (34,6%)**	**30 (28,8%)**	**32 (30,8%)**	**6 (5,8%)**
Procedures per year	< 25	25–70	> 70	
	**52 (50%)**	**33 (31,7%)**	**19 (18,3%)**	
Surgical approach	Anterior	Antero-lateral	Direct lateral	Postero-lateral
	**9 (8,7%)**	**21 (20,2%)**	**28 (26,9%)**	**46 (44,2%)**

*TR* Trauma surgeon, *RS* Reconstructive surgeons, *OP* Orthopedic physicians

**Table 5:** Answers to Sect. "Materials and methods"

Preoperative evaluation				
How do you measure LLD clinically?	U-MM distance	ASIS-MM distance	Standing with graduated blocks	Others
	**5 (4,8%)**	**87 (83,7%)**	**8 (7,7%)**	**4 (3,8%)**
How do you measure LLD clinically?	LT-BIS	LT-IT	Standing long-leg X-rays	Others
	**32 (30,8%)**	**29 (27,9%)**	**39 (37,5%)**	**4 (4%)**
Do you execute templating?	Never	Only for neck fractures	Only for elective surgery	Always
	**17 (16,3%)**	**0**	**29 (27,9%)**	**58 (55,8%)**
Digital or analog template?	Analog	Digital	I do not template	
	**38 (36,5%)**	**48 (46,2%)**	**18 (17,3%)**	
Do you believe surgical approach can influence final LLD?	Si	No		
	**40 (38,5%**	**64 (61,5%)**		

*LLD* Leg length discrepancy, *U-MM* Umbilicus–medial malleolus, *ASIS-MM* Anterior–superior iliac spine–medial malleolus, *LT-BIS* Lesser trochanter–bisischiatic line, *LT-IT* Lesser trochanter–interteardrop line

**Table 6:** Answers to Sect. "Results"

Intraoperative evaluation					
Which one of these anatomical landmarks do you use to control intraoperative lengthening?	Comparison with contralateral leg	Comparison with preoperative template measurements	Distance between lesser trochanter and tip of the trial stem	Distance between great trochanter and tip of trial stem	Others
	**40 (38,5%)**	**25 (24%)**	**27 (26%)**	**10 (9,6%)**	**2 (1,8%)**
Do you execute intraoperative X-rays?	No	Yes	Only when in doubts		
	**51 (49%)**	**35 (33,7%)**	**18 (17,3%)**		
After reduction with trial components, the prosthesis appears unstable. What do you do?	Cup evaluation (version, inclination)	Implant of longer head and accept eventual LLD	Implant of lateralizing neck of the stem, increasing femoral offset	Implant of the stem, few millimeters floating, increasing OF and LLD (especially cemented stems)	
	**24 (23,1%)**	**19 (18,3%)**	**49 (47,1%)**	**12 (11,5%)**	
Acceptable LLD at the end of operation	< 5 mm	5–10 mm	10–20 mm	> 20 mm	
	**37 (35,6%)**	**63 (60,6%)**	**4 (3,8%)**	**0**	

*LLD* Leg length discrepancy, *OF* Offset

Table 7 represents subgroups division on the basis of surgeon’s profile. We demonstrated statistically significant results for the following questions: Which “method on X-ray to measure LLD” based on years of expertise (*p* = 0.008); “execution of digital preoperative template” based on surgeon’s age (*p* = 0.001) and hospital level (*p* = 0.026); “intraoperative landmarks used to check LLD” based on number of procedures per year (*p* = 0.020); and “intraoperative X-rays” based on years of experience (*p* = 0.002) and surgical approach (*p* < 0.001) (Table 8).

**Table 7:** Subgroups division following answers to Sect. "Background"–Surgeon’s Profile

Age	** < 35 yo**	** > 45 yo**	
	39	47	
Years of experience	** < 10**	** > 10**	
	51	53	
Hospital of provenience	University hospital	Others	
	42	62	
Area of expertise	Trauma surgeon	Orthopedic physicians	Recon surgeons
	36	32	30
THA/year	** < 25**	** > 25**	
	52	52	
Surgical approach	Anterior-based	Postero-lateral	
	49	46

**Table 8:** Comparison between subgroups, statistically significant results

	< 10 years of experience				> 10 years of experience				
Preoperative X-ray measure	LT-BIS	LT-IT	FL	Other	LT-BIS	LT-IT	FL	Other	*p*
	22 43.1%	13 25,5%	14 27,4%	2 0.04%	7 13.2%	19 35.8%	25 47.2%	2 0.04%	0.008

	< 35 yo			> 45 yo			
Digital vs. analog planning	Analog	Digital	No planning	Analogic	Digital	No Planning	*p*
	4 10.2%	29 74.3%	6 15.4%	27 57.4%	13 27.6%	7 14.9%	0.001
	University hospital			Others			
	10 23.8%	26 61.9%	6 14.3%	28 45.16%	22 35.5%	12 19.3%	0.026

	< 25 THA/y				> 25 THA/y				
Intraoperative landmarks	1	2	3	4	1	2	3	4	*p*
	20 38.5%	1 0.02%	14 26.9%	17 32.7%	20 40%	9 18%	13 26%	8 16%	0.020

	< 10 years of experience			> 10 years of experience			
Intraoperative X-ray	No	Yes	If in doubt	No	Yes	If in doubt	*p*
	16 31.4%	24 47.0%	11 21.6%	35 66.0%	11 20.7%	7 13.2%	0.002
	Anterior-based			Postero-lateral			
	No	Yes	If in doubt	No	Yes	If in doubt	p
	17 34%	23 46.9%	9 18.3%	32 69.6%	6 14.3%	8 19%	< 0.0001

1 = Comparison with contralateral leg. 2 = Comparison with preoperative planning. 3 = Distance LT–Stem tip. 4 = Distance–Stem tip of GT

*LT-BIS* Lesser trochanter–bisischiatic line; *LT-IT* Lesser trochanter–interteardrop line

## Discussion

**Fig. 1:**
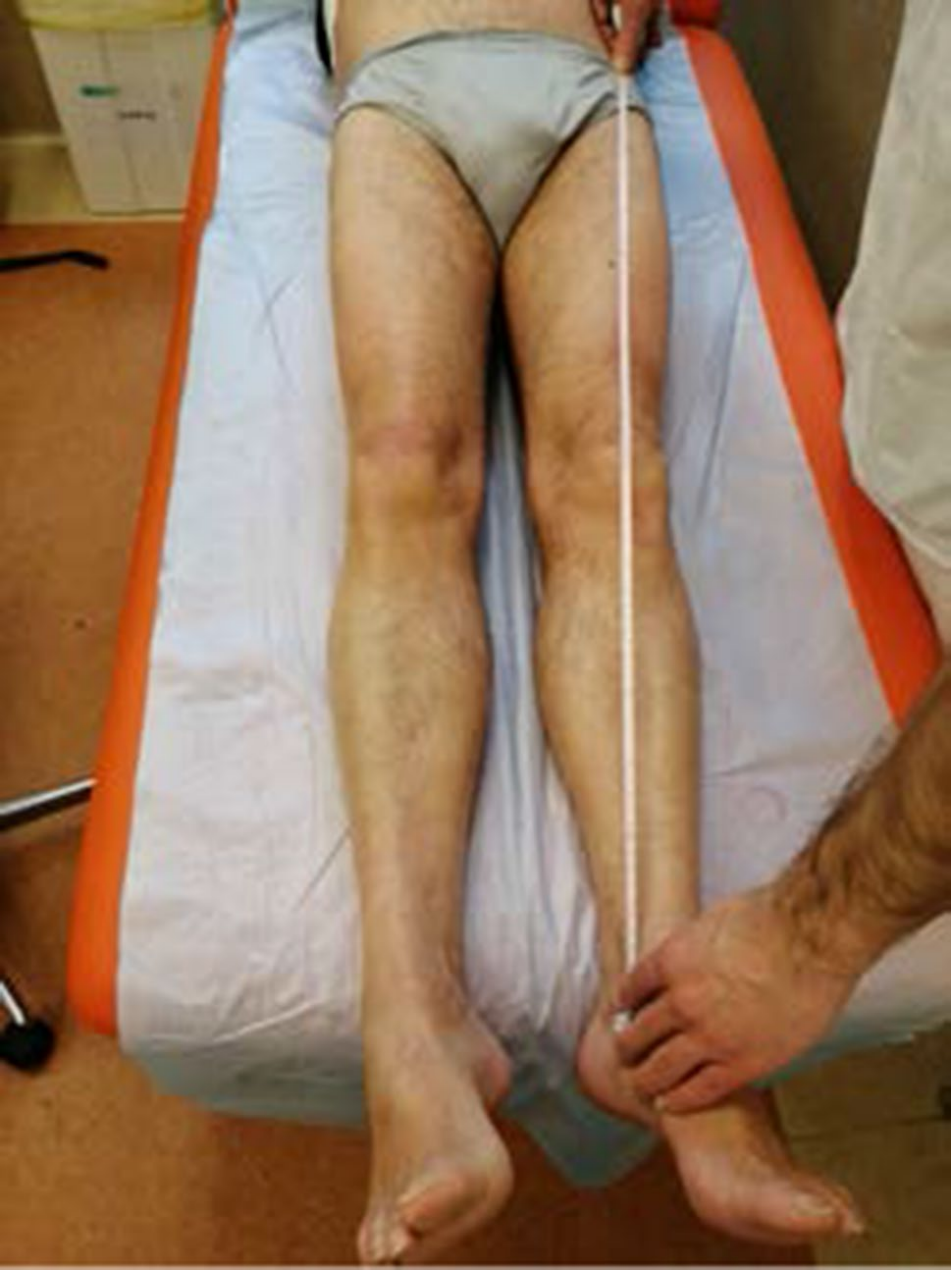
Anterior–superior iliac spine–medial malleolus (ASIS-MM) distance for clinical measure of LLD

**Fig. 2:**
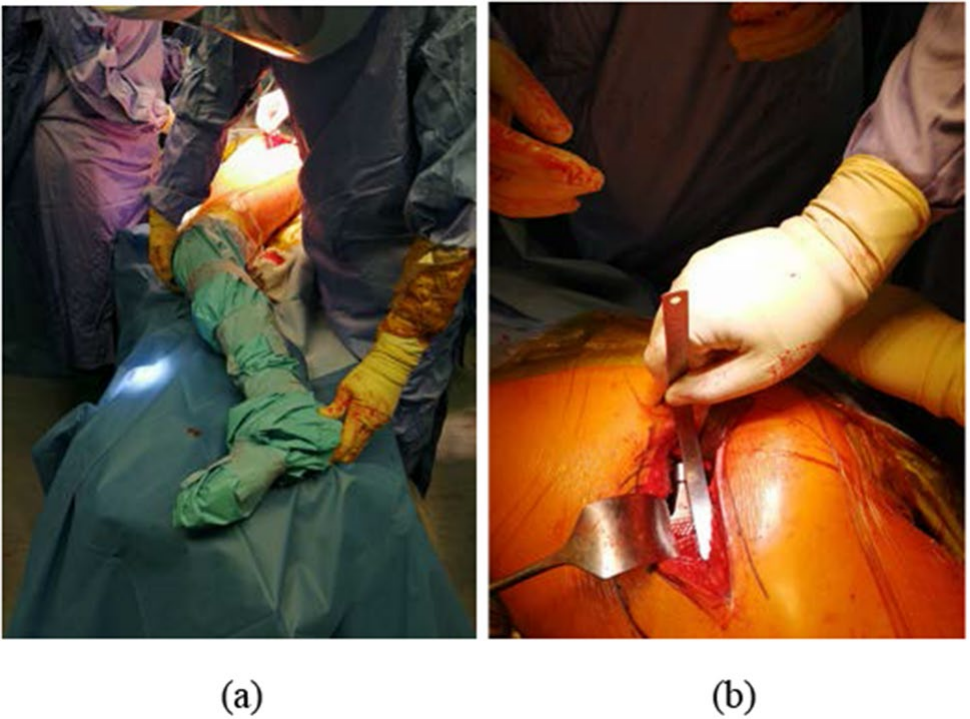
Examples of intraoperative measure of LLD: **a** comparison with contralateral leg; **b** measure of the distance lesser trochanter–tip of stem

According to Paley [9] and Glassman [10], the distance ASIS-MM is accurate and reproducible, and it is the most diffuse technique to clinically measure LLD as our results demonstrate (Fig. 1). Measurement of LLD on X-rays is more controversial. In the literature, different methods are described, and none is defined as gold standard. McWilliams [11] states the LT-BIS is the most reliable. Meermans [12], on the contrary, assumes that the distance LT-IT should be used because it is less influenced by pelvic rotation. Standing long-leg X-rays are accurate and reproducible but not available everywhere [10, 13]. Probably, the difference we found is due to change in preoperative X-ray protocols, so younger surgeons are not used anymore to measure LLD on standing long-leg X-rays. Preoperative templating [10, 14–17] is important to plan position of components in order to restore center of rotation, offset, and limb length. We demonstrated that young surgeons use digital software to template much more than older colleagues (74.3% vs. 27.6%) probably because they are more practical with the use of computers and digital software. In university hospitals, digital templating is more diffuse. The presence of residents is probably one of the main reasons, but it may also be due to lower budget in smaller hospitals to buy expensive software for digital templating. Use of intraoperative anatomical landmarks is pivotal to avoid mistakes. In the literature, they describe numerous techniques [10, 14–17]. These are the most common: comparison with contralateral leg, measure of the distance between tip of the trial stem and lesser trochanter or between tip of trial stem and greater trochanter. Many surgeons compare intraoperative findings with preoperative measures obtained from templating (Fig. 2). Results of our survey confirm there is wide difference in methods used. After performing the Chi-square test, we can state that surgeons who perform more than 25 THA per year respond differently from other surgeons. However, none of the methods were found to be predominant in either group. Use of intraoperative X-rays is well accepted and diffuse. It allows control of components positioning and LLD [10, 15, 18]. However, it is time-consuming, it gives exposure to ionizing radiations, and it can be source of contamination of the operative field. We demonstrated indeed that surgeons that prefer anterior-based approaches in supine position use more frequently intraoperative X-rays. Actually, for them, the procedure is quicker and has less potential of field contamination. Moreover, we demonstrated statistically significant difference in the use of intraoperative X-rays based on years of experience but not on number of procedures per year, so we believe that even dedicated recon surgeons find useful execution of intraoperative X-rays. From results of our survey, surgical approach does not seem to determine differences in LLD; however, Di Martino et al. demonstrated an increased risk of LLD in obese patients in which direct anterior approach was used [19].

## Conclusions

Leg length discrepancy after THA is common, but orthopedics community does not agree on how to manage it. There is wide difference in the clinical approach before surgery. During surgery, personal experience determines techniques used to avoid LLD more than the literature, and actually, none of those found in the literature appears to be better than the others. We could state that reproducibility and surgeon’s confidence with a method are more important than the method itself. Limit to this study is number of participants and small number of centers involved. Our aim in future is to open the questionnaire to national and international colleagues.

## Data Availability

https://docs.google.com/forms/d/1ZtDSypLqPxorwv8CUWFFMQGkdEQyprRmycQpV198ygc/edit#responses
